# Quality Assessment of Wild and Cultivated Green Tea from Different Regions of China

**DOI:** 10.3390/molecules26123620

**Published:** 2021-06-13

**Authors:** Maciej Chowaniak, Marcin Niemiec, Zhiqiang Zhu, Naim Rashidov, Zofia Gródek-Szostak, Anna Szeląg-Sikora, Jakub Sikora, Maciej Kuboń, Salimzoda Amonullo Fayzullo, Usmon Mamur Mahmadyorzoda, Agnieszka Józefowska, Andrzej Lepiarczyk, Florian Gambuś

**Affiliations:** 1 Department of Agroecology and Crop Production, Faculty of Agriculture and Economics, University of Agriculture in Krakow, Al. Mickiewicza 21, 31-120 Krakow, Poland; a.lepiarczyk@ur.krakow.pl; 2 Department of Agricultural and Environmental Chemistry, Faculty of Agriculture and Economics, University of Agriculture in Krakow, Al. Mickiewicza 21, 31-120 Krakow, Poland; marcin1niemiec@gmail.com (M.N.); rrgambus@cyf-kr.edu.pl (F.G.); 3 Department of Agricultural Resources and Environment, College of Tropical Crops, Hainan University, Renmin Avenue, Haikou, Hainan Province 570228, China; zqzhu@hainanu.edu.cn; 4 Department of Food Products and Agrotechnology, Polytechnical Institute of Tajik Technical University by Academician M.S. Osimi in Khujand, Lenin St. 226, Khujand 735700, Tajikistan; naimrashidov-tj@mail.ru; 5 Department of Economics and Enterprise Organization, Cracow University of Economics, 31-510 Krakow, Poland; grodekz@uek.krakow.pl; 6 Faculty of Production and Power Engineering, University of Agriculture in Krakow, 30-149 Kraków, Poland; anna.szelag-sikora@urk.edu.pl (A.S.-S.); Jakub.Sikora@urk.edu.pl (J.S.); Maciej.Kubon@urk.edu.pl (M.K.); 7 Eastern European State College of Higher Education in Przemyśl, Książąt Lubomirskich 6, 37-700 Przemyśl, Poland; 8 Ministry of Agriculture of the Republic of Tajikistan, 5/1 Shamsi Street, Dushanbe 734003, Tajikistan; a.faizullozoda@mail.ru; 9 Tajik Agrarian University Named After Shirinsho Shotemur, Rudaki Avenue 146, Dushanbe 734003, Tajikistan; m.usmon@mail.ru; 10 Department of Soil Science and Agrophysics, Faculty of Agriculture and Economics, University of Agriculture in Krakow, Al. Mickiewicza 21, 31-120 Krakow, Poland; agnieszka.jozefowska@urk.edu.pl

**Keywords:** wild tea, green tea, health risk, total phenolic content, caffeine, microelements, macroelements, oxalates, nitrates, nutritional requirements, management

## Abstract

Natural products have always enjoyed great popularity among consumers. Wild tea is an interesting alternative to tea from intensive plantations. The term “wild tea” is applied to many different varieties of tea, the most desirable and valued of which are native or indigenous tea plants. Special pro-health properties of wild tea are attributed to the natural conditions in which it grows. However, there are no complex studies that describe quality and health indicators of wild tea. The aim of this research was to evaluate the quality of wild and cultivated green tea from different regions of China: Wuzhishan, Baisha, Kunlushan, and Pu’Er. The assessment was carried out by verifying the concentration of selected chemical components in tea and relating it to the health risks they may pose, as well as to the nutritional requirements of adults. Wild tea was characterized by higher micronutrient concentration. The analyzed teas can constitute a valuable source of Mn in the diet. A higher concentration of nitrates and oxalates in cultivated tea can be associated with fertilizer use. The analyzed cultivated tea was a better source of antioxidants with a higher concentration of caffeine. There were no indications of health risks for wild or cultivated teas.

## 1. Introduction

Tea is one of the most popular beverages in the world. Its health benefits are well documented [1,2], and various studies have proven that tea consumption could have both positive and negative effects on human health [2,3,4,5]. Tea may be an important source of essential major dietary inorganic macro- and microelements. It has also been found that tea plants can accumulate large amounts of aluminum and selected heavy metals such as lead, cadmium, and arsenic [6,7]. During brewing, both essential mineral elements and toxic metals, which may pose health risks, are released into the infusion. Another aspect of tea evaluation in the context of both its dietary value and its potential health risks is the presence of inorganic and organic chemical compounds. In terms of permanent intake, the most common undesirable chemical compounds in tea infusions include oxalates, which are connected with the risk of renal and urethral calculus formation, and nitrates, which may increase the risk of cancer. Conversely, organic compounds present in tea, such as phenolic compounds, flavones, and compounds that determine the antioxidant properties of tea, constitute desirable constituents of tea and contribute to its anticarcinogenic properties. Among the organic compounds in tea, caffeine is an important qualitative indicator, which, on the one hand, is a desirable compound; however, on the other hand, due to its action when a certain limit is exceeded, it can pose a health risk. According to some authors [4,8], tea can be an important source of these organic compounds as part of the daily diet. The chemical composition of tea determines its quality and is dependent on many factors. These include habitat conditions, such as the type of soil, sun exposure, temperature, and precipitation, as well as cultivation technology, nutrient supply, and plant density [9,10,11,12,13]. Most of the teas available come from plantations with different cultivation intensities. The teas available can be divided not only according to the variety, method of production, and region of origin, but also by the method of harvesting the tea for mass production. Tea is obtained not only from agroecosystems but also from agro-forest and forest ecosystems [14]. Forest agroecosystems and forest ecosystems are recognized as collection locations for “wild tea”. However, the term “wild tea” is ambiguous; it is applied to a wide variety of teas and can refer to (1) tea plants grown on plantations with minimal pruning and without fertilization and pesticides, (2) tea plants grown in forests, also with minimal pruning and without fertilization and pesticides, and (3) tea plants in abandoned and feral plantations covered by the natural expansion of the forest ecosystem. The most desirable and valued teas are native or indigenous plants, including plants growing in forests naturally, with the only human interference in their development being collection. The term “wild tea” also refers to ancient tea trees growing in the abovementioned conditions [15]. Tea plants are thought to originate from southwestern China, including Yunnan and the adjacent provinces. However, the wild progenitor of tea has not yet been found [16]. The research to date [16,17] shows that, compared with wild species, cultivated germplasms usually show lower levels of genetic diversity because breeding practices tend to reduce genetic diversity to a greater extent than domestication. Special pro-health properties of wild tea are attributed to the natural conditions in which it grows. However, there are no studies that describe the quality and health indicators of wild tea. Ensuring food safety and commercial quality in accordance with industry guidelines requires the collection of data regarding the relationship between production technology and specific features of products such as chemical composition [11,12].

The aim of this research was to evaluate the quality of wild and cultivated teas collected in different regions of China. The quality assessment was carried out by verifying concentrations of selected chemical components in teas and relating them to the health risks they may pose, as well as to the nutritional requirements of adults.

## 2. Results and Discussion

### 2.1. Microelements

After water, tea is the most consumed beverage in the world, making it a major dietary source of different nutrients [5]. Tea samples were collected from tea plantations (cultivated tea) and forests (wild tea) from four different regions in China: Wuzhishan, Baisha, Kunlushan, and Pu’Er. Information about sampling locations and agrochemicals used on plantations is presented in Table 1.

The mean concentrations of microelements in wild and cultivated dry tea material from different regions are presented in Table 2. The metal concentrations in the studied dry tea material could be ranked in decreasing order as Al > Mn > Fe > Ba > Zn > Cu > Ni > Pb > Se > Li > Cd > As > Co for wild tea and Al > Mn > Fe > Ba > Cu > Zn > Ni > Pb > Se > Li > Cd > Co > As for cultivated tea. Significant differences in concentrations of microelements in dry tea material were observed for Al, Cu, Ni, and Zn. On average, higher concentrations of Al and Ni were determined in wild dry tea material compared to cultivated tea, with values 32.4%, 34.4%, and 54.0% higher, respectively, than for cultivated tea. Cu concentration was on average 17.2% higher in cultivated dry tea material than it was in wild tea. For other microelements, differences were not statistically significant. According to Street et al. [18], the total microelement components in tea depend on many factors, primarily, the age of the tea leaves, as well as the soil conditions, rainfall, altitude, genetic makeup of the plant, etc. In the present study, the deliberate selection of the tea samples tested, through the collection of leaves of a similar age and the selection of neighboring regions of plant occurrence, allowed us to standardize the studied population and reduce the number of random factors influencing the variability. Due to the much higher yield per unit area of tea grown, the lower concentrations of micronutrients in cultivated plants may be related to the effects of microelement dilution in biomass, but only in situations when fertilization with the microelements was not used [19]. The higher concentration of copper and zinc in the cultivated dry tea material can be related to the use of pesticides containing copper oxychloride and foliar fertilizers with *zinc sulfate* in its cultivation [20]; cultivated tea in the present research came from a plantation where producers used pesticides with copper two to three times per year and a minimum of one treatment with zinc sulfate.

The approval of a food product on the market is related to the fulfillment of certain requirements, and these requirements are different in different regions of the world. With respect to microelement concentrations in tea, various legal regulations cover several elements, the verification of which is considered crucial due to their harmfulness. The results of our experiments show that the concentrations of Pb, As, and Cu in all tea samples were lower than the corresponding thresholds set by the WHO [21] and Chinese national food safety standards for maximum levels of contaminants in foods [22,23]. In the case of cadmium, the concentration of this element in dry tea material collected from the C1 region was 0.33 mg·kg^−1^. According to the WHO [21], the limit for Cd concentration in dry tea material is 0.30 mg·kg^−1^. However, there is no regulation on metal concentration in teas for the remaining analyzed metals and Al. Because of the lack of information about acceptable concentration levels in tea for other evaluated microelements, the Oral Reference Dose index (RfD) was used for the assessment of health risks related to tea intake. For health risk evaluation, the concentration of microelements in tea infusions was analyzed. The concentrations of microelements in the infusions are presented in Table 3. The wild and cultivated tea infusions did not differ significantly in terms of the elemental concentration sequence, which was as follows: Al > Mn > Fe > Ba > Ni > Cu > Zn > Cr > Pb > Li > Se > As > Co > Cd. The infusions of wild and cultivated teas differed significantly with respect to Al, Ba, Cd, and Ni. The highest concentration of Al was observed in the W4 tea infusion, with the lowest in C2; in wild tea infusions, the concentration of Al on average was 21.9% higher than in infusions from cultivated tea. Moreover, concentrations of Ba and Ni in infusions from wild teas were higher by 18.6% and 68.0%, respectively, than in those from cultivated teas. In turn, higher concentrations of Cd were found in infusions of cultivated teas by 17.6%. The obtained results concerning the concentration of elements in tea infusions are comparable to the results obtained by other authors [7,18,24,25,26].

The results show that there is a wide variation in the percentage transfer of the examined elements from the dry tea materials to the infusions (Table 4). With respect to the transfer index for micronutrients, there were no significant differences between wild and cultivated tea. The sequence of extraction and percentage transfers of the elements were, in decreasing order, As > Ni > Pb > Li > Co > Cu > Al > Zn > Cd > Mn > Ba > Fe (in terms of means for wild and cultivated tea). The solubilities of As, Ni, Li, Pb, and Co were the highest among the elements studied (21.8–35.3%), whereas Fe and Ba were insoluble, and only a small portion of their trace metal content could leach into an infusion (average 5.4–6.3%). The solubilities of Mn, Cd, Fe, Zn, Al, Cu, and Cr were similar, ranging from 8.3% to 14.8%. The sequence of extraction and percentage transfers of all elements except for Mn and Zn were similar to those noted in cultivated tea [8,27,28,29,30].

The calculated EDI values (mg·kg^−1^ bw·day^−1^) of microelements through tea infusion intake are listed in Table 5. It can be seen that the average EDI values (mg·kg^−1^ bw·day^−1^) of Al, As, Ba, Co, Li, Mn, Ni, and Pb for the wild tea infusion were greater than those for the cultivated tea infusion. In contrast, the average EDI of Cd, Cu, Fe, and Zn from wild tea infusion intake was lower than that from cultivated tea infusion intake. Using the values obtained from calculating EDI and RfD, the THQ index was calculated. This indicator allowed us to verify the health risk associated with the intake of selected elements. The calculated THQ values are listed in Table 6. Higher concentrations of Al, As, Ba, Co, Li, Mn, Ni, Pb, and Se in the infusion from wild tea translated into a higher value of the THQ index. The aforementioned Cd exceedance in the C1 dry tea material did not affect the RfD exceedance. This was due to the limited transfer of ingredients from the dry material into the infusion, which was 6.9% on average for C1 tea. THQs of individual metals were all less than one, suggesting that none of the heavy microelements results in adverse health effects for adults via daily tea intake, which makes the tea from the analyzed regions of China safe for consumers. The HI index was also less than one, which indicates no risk related to the concentration of micronutrients in the tested tea infusions. Regarding HI index, there were no significant differences between wild and cultivated tea. Compared with other foodstuffs, it has been found that the HI value of metals for tea infusions is higher than for traditional Chinese egg products [31] and infusions of herbal flowers [32], but lower than for rice and vegetables [32,33]. In addition to the health risk indicators, the values of the daily requirement for selected micronutrients and their fulfillment by intake with tea are presented (Table 7). Micronutrient intake of Cu, Fe, and Zn with the infusion accounted for around 0.2% to 1.7% of the daily requirement and was greater for cultivated tea. In the case of Mn, intake of this element from wild tea was 125.1% of the average daily requirement and 120.4% for cultivated tea, which did not exceed a tolerable upper intake level of 11 mg·day^−1^.

When describing the health aspects of micronutrient intake, several important elements should be taken into account. Other sources of heavy metals in the dietary structure, such as drinking water, vegetables, fruits, and grains, combined with tea, may play important roles in forming probable health risks. The presented microelements that enter the human body are not all taken up by the gastrointestinal tract. According to Zhang et al. [3] and Laparra et al. [33], the bioavailability of microelements absorbed accounts for about 40%. Moreover, the microelement bioavailability to humans is impacted by many factors, such as individual lifestyle, food type, and the dose taken up by the human digestive system. The bioavailability of heavy metals may differ according to meals and mealtimes [33].

### 2.2. Macroelements

The concentrations of Ca, K, Mg, Na, and P are presented in Table 1. In this study, the most abundant element among the macroelements was K, followed by Ca, Mg, P, and Na for wild and cultivated tea. Significant differences were observed for P concentration. Higher P concentrations were found in cultivated tea (dry material) by 4.3–29.2%. The concentration of elements in the leaves influenced their concentrations in the infusions. The only change was a significantly higher mean Ca concentration in wild tea infusions compared to cultivated tea infusions (Table 5). As in the case of microelements, the main sources of elements in tea plants and leaves are their growth media (i.e., soils and their characteristics, fertilization). In dry tea material from cultivated plantations, the higher contents of P resulted from the use of NPK fertilizers.

Average extraction rates were the highest for Na and K according to the classification presented by Szymczycha-Madeja et al. [28], and these elements can be classified as moderately extractable. In turn, the remaining macroelements can be classified as poorly extractable, with the lowest transfer factor calculated for Ca (Table 6). It was observed that the transfer coefficient was significantly higher for Na and P in cultivated tea and Ca for wild tea. The obtained results concerning the concentrations of macronutrients in the dry material and solutions for both wild and cultivated teas are comparable to the results presented by other authors, which concern teas obtained from plantations characterized by different degrees of cultivation and fertilization, as well as plantations without fertilization [18,24,28,29].

The daily intake of macroelements from tea was evaluated in view of the latest recommended dietary intakes of the Institute of Medicine [34]. Daily consumption of tea results in intakes of Ca, K, Na, Mg, and P in the range 0.02–2.01% of their respective recommended dietary allowances or adequate intakes (Table 7). Similar results for different variations of cultivated tea were obtained by other authors [24,25,29].

### 2.3. Nitrates and Oxalates

**Figure 1:**
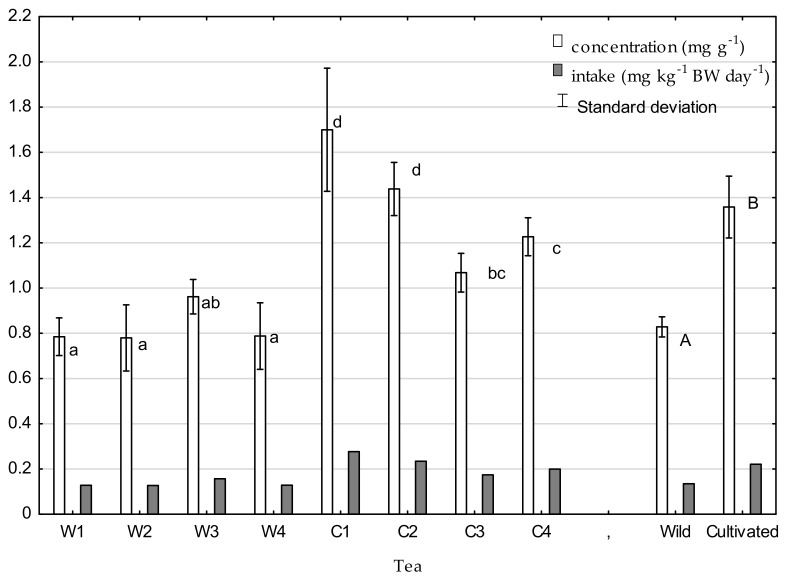
Nitrate concentrations in tea and daily intake with tea infusion. Means with different letters are significantly different, according to Tukey’s test (*p* ≤ 0.05). W1wild tea, Wuzhishan; W2-wild tea, Baisha; W3-wild tea, Kunlushan; W4-wild tea, Pu’Er; C1-cultivated tea, Wuzhishan; C2-cultivated tea, Baisha; C3-cultivated tea, Kunlushan; C4-cultivated tea, Pu’Er.

**Figure 2:**
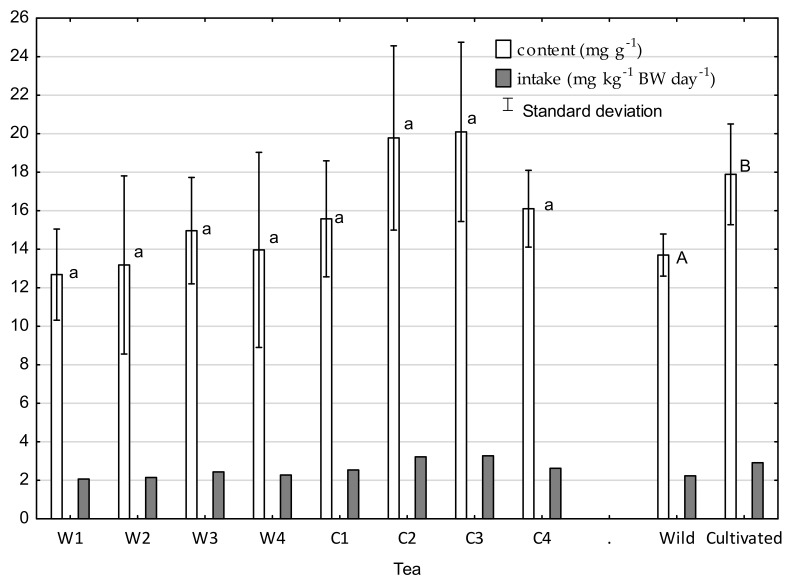
Oxalate concentration in tea and daily intake with tea infusion. Means with different letters are significantly different, according to Tukey’s test (*p* ≤ 0.05). W1-wild tea, Wuzhishan; W2-wild tea, Baisha; W3-wild tea, Kunlushan; W4wild tea, Pu’Er; C1-cultivated tea, Wuzhishan; C2-cultivated tea, Baisha; C3-cultivated tea, Kunlushan; C4-cultivated tea, Pu’Er.

The concentration of nitrates in the tested tea ranged from 0.37 to 1.11 mg·g^−1^ for wild tea and from 0.95 to 2.28 mg·g^−1^ for cultivated tea. A significantly higher concentration of nitrates in cultivated tea resulted from nitrogen fertilizers used in its cultivation. The calculated daily EDI values are presented in Figure 1. The acceptable daily intake (ADI) for nitrate was determined by the European Commission’s Scientific Committee on Food [35] and ranges from 0 to 3.7 mg·kg^−1^ of body weight per day, which is equivalent to the intake of 259 mg nitrate/day for an adult weighing 70 kg. In the present research, average daily intake of nitrates accounted for only about 3.7% of the ADI in the case of wild tea and 6.1% in the case of cultivated tea. Previously, nitrate was considered a precursor of the *N*-nitroso compound that was classified as a human carcinogen. The International Agency for Research on Cancer (IARC) concluded that there was no substantial evidence implicating nitrates as animal carcinogens in 2010 [36]. Moreover, in recent epidemiological investigations, dietary nitrates showed no association with gastric cancer or esophageal cancer in humans [37,38]. While the determination of RfD in infants is associated with the occurrence of methemoglobinemia, the effect of nitrates on the adult body is ambiguous. The results of research on the oxalate concentration in tea infusions are presented in Figure 2. The concentrations of oxalates in cultivated tea were significantly higher than in infusions made with wild tea, ranging from 7.8 to 18.5 mg·g^−1^ in wild tea infusion and 13.1 to 23.1 mg·g^−1^ in cultivated tea. Morita et al. [39] reported that one of the factors determining the oxalate content in tea is the form of nitrogen fertilization and its amount. Oxalate synthesis is suppressed under a lower nitrate content since oxalate is synthesized to neutralize OH^--^ produced via nitrate reduction. On the plantations from which tea samples were taken, ammonium nitrate was used. Oxalate intake was assessed in view of recommendations by the American Dietetic Association, according to which the levels of oxalate for people with increased risk of kidney stones should not exceed 40–50 mg per day (of bioavailable compound). With an average intake of 11.4 g per day and average bioavailability of oxalate of 9% [40], 14.1 mg of oxalate would be absorbed from wild tea (total intake: 156.2 mg per day); in the case of cultivated tea (total intake: 203.9 mg per day), 18.3 mg of oxalate would be absorbed. Therefore, it can be concluded that consumption of the presented cultivated teas (fertilized with nitrate) is more dangerous for people with a risk of kidney stones than drinking the same amount of wild tea. However, for both teas, the risk is below alarming levels.

### 2.4. Caffeine, Antioxidant Capacity, and Total Phenolic Content

**Figure 3:**
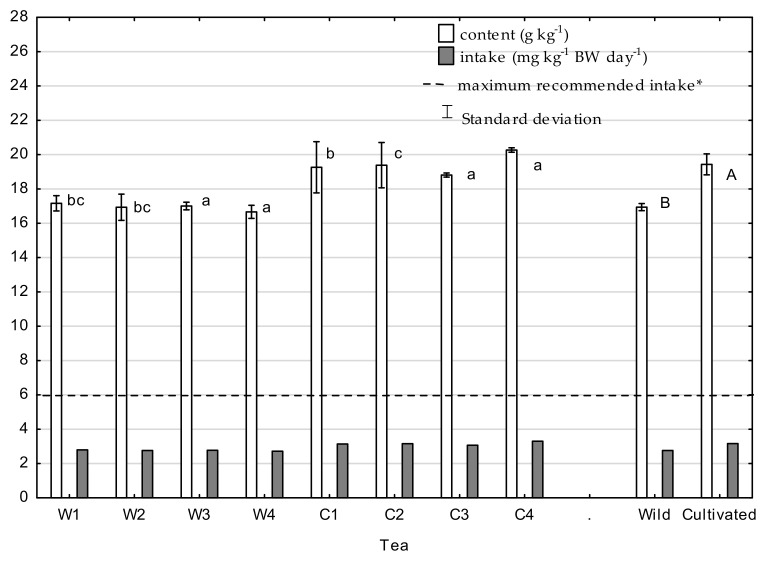
Caffeine content in tea and daily intake with infusion; * maximum recommended intake not associated with adverse effects such as general toxicity (6 mg·kg^−1^ bw·day^−1^) [27,45]. Means with different letters are significantly different, according to Tukey’s test (*p* ≤ 0.05). W1-wild tea, Wuzhishan; W2-wild tea, Baisha; W3-wild tea, Kunlushan; W4-wild tea, Pu’Er; C1-cultivated tea, Wuzhishan; C2-cultivated tea, Baisha; C3-cultivated tea, Kunlushan; C4-cultivated tea, Pu’Er.

Caffeine concentrations in tea infusions, as well as daily intake and maximum recommended intake, are presented in Figure 3. The caffeine concentration in tea is one of the determinants of its quality. The concentration of caffeine in cultivated tea infusions was significantly higher than in infusions made with wild tea; for cultivated tea, it ranged from 18.2 to 20.4 g·kg^−1^, and, for wild tea, it ranged from 16.4 to 17.5 g·kg^−1^. These values are similar to those reported for tea by other authors. Bae et al. [41] reported results of caffeine levels in cultivated teas, 20.50 mg·g^−1^ in green tea and 22.21 mg·g^−1^ in black tea, while Tfouni et al. [42] reported 9.9–17.0 mg·g^−1^ for green tea and 10.8–24.1 mg·g^−1^ for black tea. In the present research, the higher concentration in cultivated tea was caused by the usage of NPK fertilizers. According to Ruan [43], the caffeine concentration in tea is influenced by the level and form of N supply to tea plants, and its increase is directly proportional to the increasing dose of N-fertilizer. Hajiboland [34] also stated that the application of fertilizers as a combination of N and K improves the caffeine content of tea leaves. With respect to nutrient-deficient plants (wild tea), their low caffeine content may be a consequence of lower N availability. Pompelini et al. [44] suggested that caffeine is degraded into theophylline and other N-products to supply N in N-deficient plants. Calculated daily intake did not exceed recommended values for adults, at a level from 44.5% to 55.3% of the maximum daily dose, i.e., 189.9–232.0 mg of caffeine/day. A comprehensive review of the effects of caffeine consumption on human health concluded that, for healthy adults, moderate chronic intake of caffeine up to 400.0 mg·day^−1^ is not associated with adverse effects on cardiovascular health, calcium balance and bone status, behavior, cancer risk, or male fertility [27,45]. The European Commission’s Scientific Committee of Food Safety Authority and Health Canada both recommend that women consume no more than 300.0 mg of caffeine·day^−1^ during pregnancy [27]. In turn, despite conflicting results regarding the association between caffeine consumption and spontaneous abortion, the American College of Obstetricians and Gynecologists recommends that pregnant women restrict their caffeine intake to less than 200.0 mg·day^−1^ [46].

**Figure 4:**
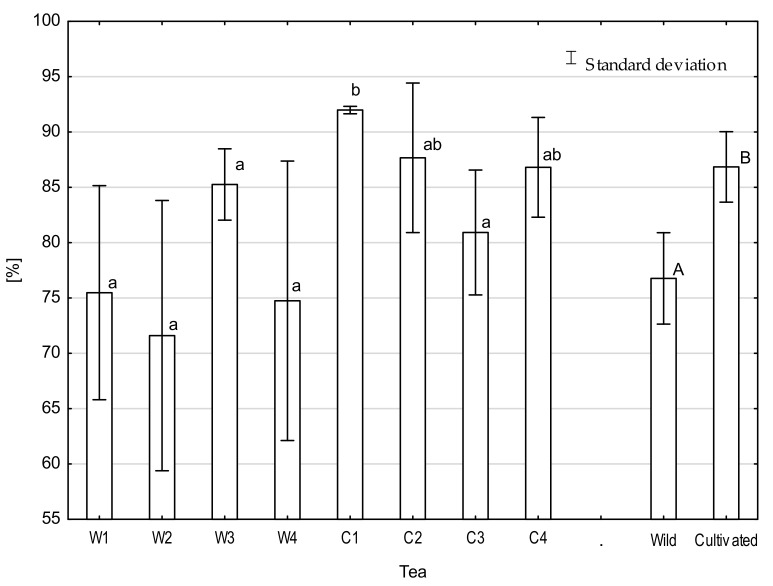
The DPPH radical-scavenging activity of analyzed tea. Means with different letters are significantly different, according to Tukey’s test (*p* ≤ 0.05). W1-wild tea, Wuzhishan; W2-wild tea, Baisha; W3-wild tea, Kunlushan; W4-wild tea, Pu’Er; C1-cultivated tea, Wuzhishan; C2-cultivated tea, Baisha; C3-cultivated tea, Kunlushan; C4-cultivated tea, Pu’Er.

**Figure 5:**
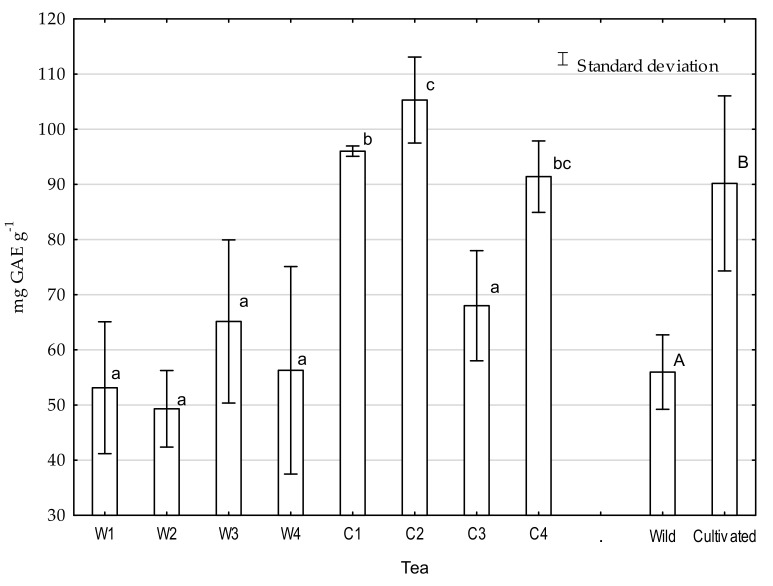
Total phenolic content in tea infusion expressed as mg gallic acid equivalent (GAE)·g^−1^ DW. Means with different letters are significantly different, according to Tukey’s test (*p* ≤ 0.05). W1-wild tea, Wuzhishan; W2-wild tea, Baisha; W3-wild tea, Kunlushan; W4-wild tea, Pu’Er; C1-cultivated tea, Wuzhishan; C2-cultivated tea, Baisha; C3-cultivated tea, Kunlushan; C4-cultivated tea, Pu’Er.

The antioxidant capacity in analyzed tea ranged from 51.5% to 92.3% and total phenolic contents from 49.6 to 113.0 mg GAE·g^−1^ (Figure 4), which is consistent with the results of different authors [47,48,49]. The antioxidant capacities and total phenolic contents of wild and cultivated teas differed significantly. The antioxidant capacities for cultivated tea were on average 11.8% higher than in wild teas, and total phenolic content was 25.5% higher in cultivated tea (Figure 5). The Pearson correlation coefficient between total phenolic and DPPH clearance was 0.72, which is consistent with previous research [49,50]. As in previous indicators, the main factor affecting differences between teas was nutrition with NPK fertilizers. According to Ruan [43], there is a positive relationship between N concentration and the accumulation of the abovementioned quality-related components of tea plants. The antioxidant capacity and phenolic compound contents are some of the most important indicators of tea quality. Phenolic compounds are included in a large group of secondary metabolites found in natural products and are characterized by a wide range of bioactivities that reduce the risk of oxidative-stress-related diseases. Among many studies, a primary focus was the evaluation of the effect of various phenolic compounds on cancer or cardiovascular risk factors [50,51]. Studies by Vitale et al. [52] on the effects of a diet containing polyphenolic compounds on cardiovascular disease found that people with the highest intakes of energy-adjusted polyphenols had more favorable cardiovascular risk factor profiles compared to people with the lowest intakes. Conversely, some research has suggested that green tea extract supplements with high doses of catechins could cause hepatotoxicity; according to Navarro et al. [53] and Mazzanti et al. [54], this is possibly due to oxidative stress caused by epigallocatechin gallate (EGCG) and its metabolites. There is a gap related to the determination of the recommended daily intake (RDI) for phenolic compounds. Due to the emerging reports about the ambiguous nature of these chemical compounds, it is necessary to define the RDI.

## 3. PCA Analysis

**Figure 6:**
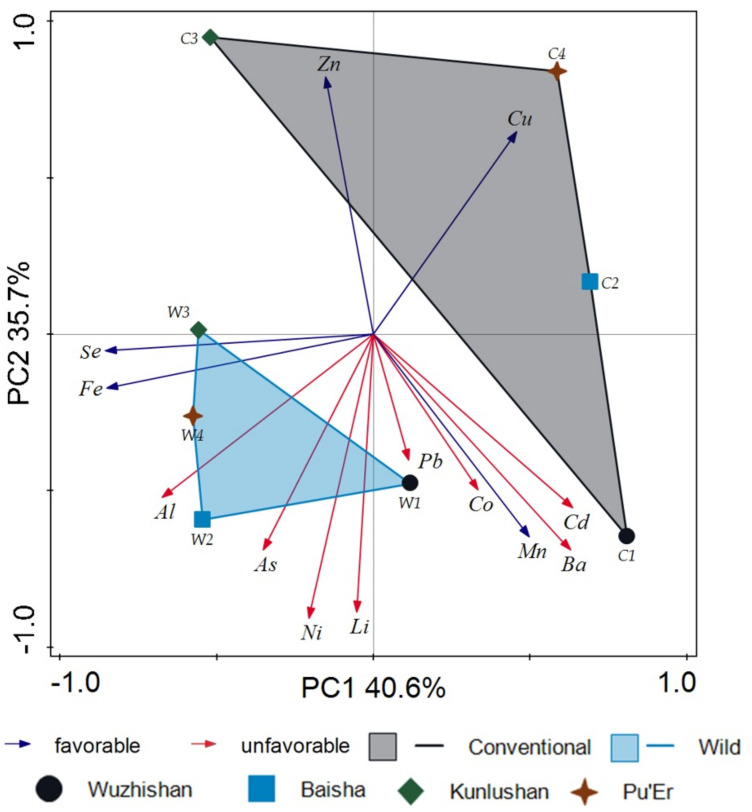
Principal component analysis (PCA) score derived from 14 microelements of 64 tea samples: W1-wild tea, Wuzhishan; W2-wild tea, Baisha; W3-wild tea, Kunlushan; W4-wild tea, Pu’Er; C1-cultivated tea, Wuzhishan; C2-cultivated tea, Baisha; C3-cultivated tea, Kunlushan; C4-cultivated tea, Pu’Er.

PCA is an effective way to discriminate between data observed [55]. It also involves a linear transformation of multiple variables into a low-dimensional space that retains the maximum amount of information about the variables [56,57]. Generally, the score plot provides a visual determination of similarity among the samples. Figure 6 shows that the score plot (score derived from 14 microelements) in the first two principal components (PC1 and PC2) represented 76.2% of the total variability for raw material. The same figure shows that types of wild tea were clearly distinguished from cultivated tea in the PCA model. The conducted analysis indicated a greater affinity of wild tea for As, Al, Cr, Fe, Ni, and Li; on the other hand, cultivated tea had a greater affinity for Cu and Zn.

**Figure 7:**
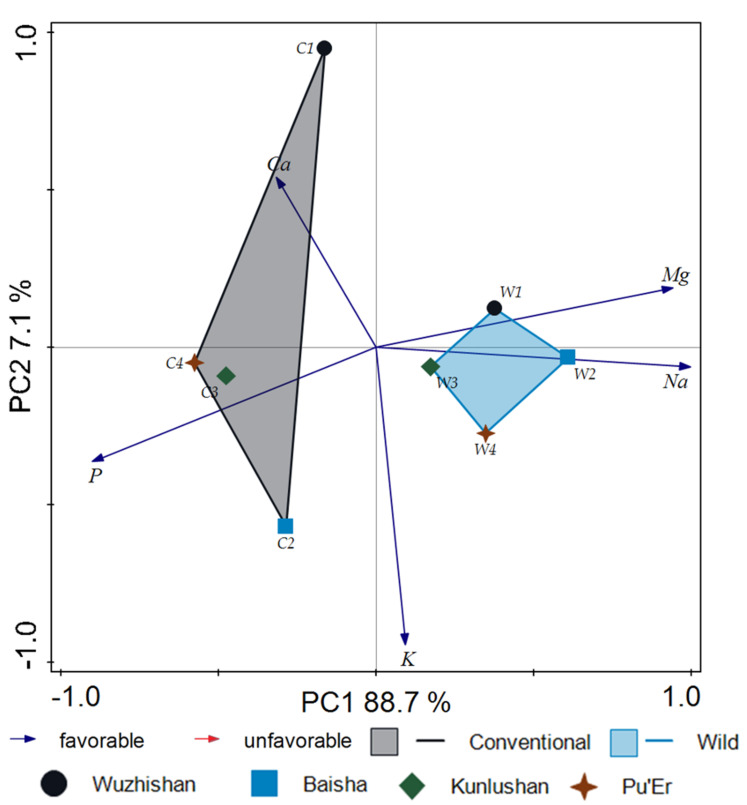
Principal component analysis (PCA) score derived from five macroelements of 64 tea samples: W1-wild tea, Wuzhishan; W2-wild tea, Baisha; W3-wild tea, Kunlushan; W4-wild tea, Pu’Er; C1-cultivated tea, Wuzhishan; C2-cultivated tea, Baisha; C3-cultivated tea, Kunlushan; C4-cultivated tea, Pu’Er.

Similar results were obtained with regard to macroelement content (Figure 7). Figure 7 shows that the score plot in the first two principal components (PC1 and PC2) represented 95.8% of the total variability. In the case of macronutrients, the PCA model also clearly distinguished between wild tea and cultivated tea. The conducted analysis showed a greater affinity of wild tea for Mg and Na, whereas cultivated tea was more related to P and Ca.

**Figure 8:**
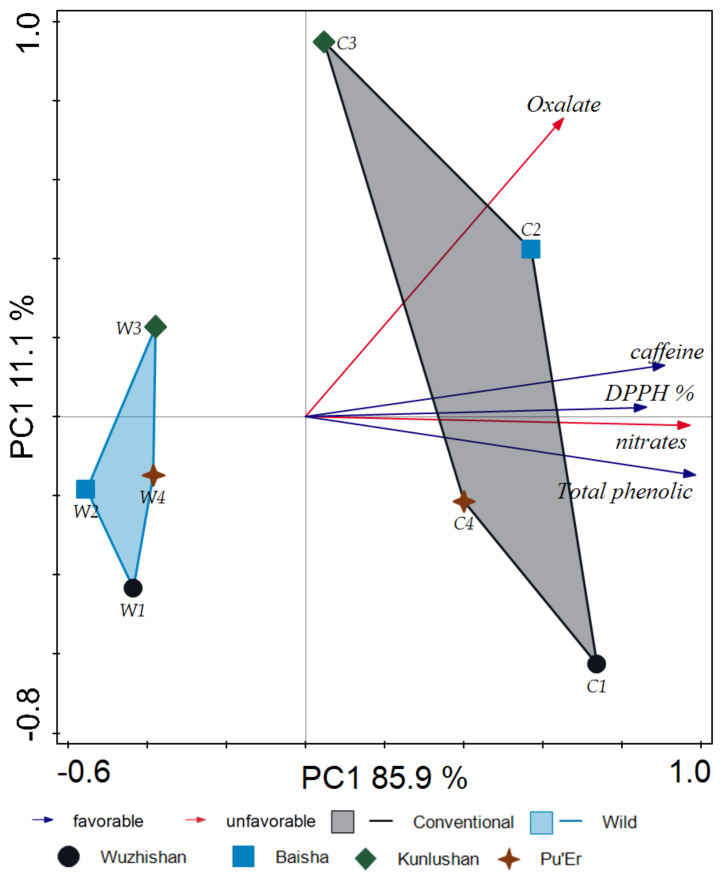
Principal component analysis (PCA) score derived from oxalates, nitrates, caffeine content, DPPH, and total phenolic content of 64 tea samples: W1-wild tea, Wuzhishan; W2-wild tea, Baisha; W3-wild tea, Kunlushan; W4-wild tea, Pu’Er; C1-cultivated tea, Wuzhishan; C2-cultivated tea, Baisha; C3-cultivated tea, Kunlushan; C4-cultivated tea, Pu’Er.

With regard to the content of oxalates, nitrates, and caffeine, as well as total phenolic content and oxidation activity, the PCA model also clearly distinguished between wild tea and cultivated samples (Figure 8). The score plot in the first two principal components (PC1 and PC2) represented 96.0% of the total variability.

## 4. Materials and Methods

### 4.1. Sample Collection and Preparation

Tea samples were collected from tea plantations (cultivated tea) and forests (wild tea) from four different regions in China: Wuzhishan, Baisha, Kunlushan, and Pu’Er (Table 1). Research locations were selected due to the high production potential of tea in the selected areas related to the supply of both cultivated and wild tea. An attempt was made to select sampling sites such that given batches of both wild and cultivated tea were collected from places with similar climatic and habitat conditions. It was assumed that the cultivated tea was from plantations of standard cultivation intensity for the region. Because of the definition of “wild tea” as a marketable product, this term may refer to several different varieties of tea, each of which is “wild” in its own way. For our experiments, *Camellia sinensis* plants growing in forests without any cultivation were selected, and the only intervention was harvesting. The locations of plants and their clusters were indicated by local collectors who were suppliers of raw materials to local manufacturers. In total, 64 samples were collected during the tea harvesting season of 2019. Tea leaves (the top four leaves) were plucked by hand at random from each site (within a 1000 m^2^ area). Each sample consisted of eight subsamples in each site. Each subsample consisted of 1 kg of tea leaves. Green tea was prepared according to the following processes: withering, pan-frying, rolling, and drying.

### 4.2. Tea Brewing

The tea infusion was performed by adding 100 mL of boiling distilled water to 3.0 g of the prepared tea sample. After 10 min brewing time, the plant matter was separated from the infusion by filtration. The infusions were cooled to room temperature, and analyses were carried out immediately [8].

### 4.3. Micro- and Macroelement Contents

The 18 elements were chosen for analysis on the basis of a literature study [2,3,5,6,7,8,18,26]. In order to determine the micro- and macroelement concentrations in the dry tea material, samples were mineralized in a mixture of nitric acid solution and dihydrogen dioxide at a volume ratio of 1:3. The weight of an analytical test portion was maximum 0.5 g of dry weight. Prepared tea infusion samples were acidified by adding 2 cm^3^ of nitric acid per 100 cm^3^ of water. The samples were concentrated fivefold, and then the concentration of elements (Al, As, Ba, Cd, Co, Cu, Fe, Li, Mn, Ni, Pb, Se, Zn, Ca, Mg, Na, K, and P) in the prepared solutions was determined by inductively coupled plasma atomic emission spectroscopy (ICP-AES), using an Optima 7600 instrument made by Perkin Elmer (Norwalk, CT, USA). The wavelengths, which were used to determine the concentrations of the tested elements and limits of determination with regard to the methods used, are presented in Table 2. To control the accuracy of elemental analyses, a certified reference material, IAEA-V-10, was used. Table 8 shows the results of reference material analyses and the recovery value on the basis of the analyses performed in three replications.

### 4.4. Nitrate Content

The nitrate content was determined by the colorimetric method with the use of salicylic acid in a sulfuric acid solution [58]. For analysis, 0.2 cm^3^ of tea infusion was prepared with 0.8 cm^3^ of salicylic acid in sulfuric acid and 18 cm^3^ of sodium hydroxide solution with a concentration of 2 M. In the solutions obtained in this way, the absorbance value was determined at a wavelength of 410 nm using a Beckman DU640 instrument (Hayward, CA, USA). All assays were performed in triplicate.

### 4.5. Oxalate Content

The oxalate content was determined by titration [59], and 10 cm^3^ of the prepared infusions were taken for analysis and transferred to a centrifuge tube with a capacity of 25 cm^3^. Then, 5 cm^3^ of calcium chloride solution and 5% acetone were added. The resulting precipitate was dissolved in a 10% sulfuric acid solution. The obtained solution was titrated with a 0.02 M permanganate solution. All assays were performed in triplicate.

### 4.6. Caffeine Content

The tea infusion was mixed with dichloromethane at a volume ratio of 25:25 mL for the extraction of caffeine. First, a mixture of the solution was stirred for 10 min. Then, using a separatory funnel, caffeine was extracted by dichloromethane from the solution. The extraction of caffeine proceeded four times with 25 mL of dichloromethane at each round. The caffeine extracted by dichloromethane at each round was stored in volumetric flasks. Finally, the absorbance of the solution was measured by a UV–Vis spectrophotometer (Beckman DU640, Hayward, CA, USA) in the range 200–500 nm against the corresponding reagent blank. All glassware was thoroughly cleaned, rinsed with distilled water, and dried before use [60,61]. All assays were performed in triplicate.

### 4.7. Antioxidant Activity of Infusion by the DPPH Method

The antioxidant activity of samples was measured with the spectrophotometric method using synthetic radical DPPH (2.2-diphenyl-1-picrylhydrazyl) [62,63]. The samples were diluted 10 times. The spectral absorbance was immediately measured at 518 nm using a UV–Vis spectrophotometer (Beckman DU640, Hayward, CA, USA). The results are expressed as inhibition of DPPH radical as a percentage. All assays were performed in triplicate.

### 4.8. Total Phenolic Content

The total phenolic content was measured using the Folin–Ciocâlteu method [62]. The absorbance was measured at a wavelength of 760 nm. Gallic acid was used as a standard, and the results are expressed as milligrams of gallic acid equivalent (GAE) per gram of DW. All assays were performed in triplicate.

### 4.9. The Estimated Daily Intake

The calculation formula for estimated daily intake (*EDI*) is

*EDI_i_* = (*C_i_* × *IR*)/(*bw* × 1000), (1)

where *C_i_* is the concentration of nutrients in the tea infusion (mg·kg^−1^), *IR* is the ingestion rate of tea leaves (11.4 g·person^−1^·day^−1^) [7], and *bw* is body weight (70 kg for adults) [5].

### 4.10. The Health Risks

The health risks to tea consumers as a result of long-term exposure to Al, As, Ba, Cd, Co, Cu, Fe, Li, Mn, Ni, Pb, Se, and Zn through tea intake were assessed on the basis of the estimated daily intake (*EDI*), target hazard quotient (*THQ*), and hazard index (*HI*) [3,8]. If the *HI* is less than one, the exposure dose is lower than the adverse reaction threshold and does not present a carcinogenic risk. If the *HI* is more than one, the exposure dose is higher than the adverse reaction threshold, and it is very likely that some microelements (heavy metals) will have negative effects on human health. When the *HI* value is more than 10.0, there is a chronic toxic effect on human health *HI* [3]. The formulas for *THQ* and *HI* are as follows:

*THQ_i_* = *EDI_i_*/*RfD_i_*, (2)

*HI* = *THQ*_1_ + *THQ*_2_ +…+ *THQ_n_*, (3)

where RfD_i_ (mg·kg^−1^ bw·day^−1^) is the oral reference dose for metal, regulated by the US Environmental Protection Agency [61], *THQ_i_* is the target hazard quotient of metal I, and HI is the total health risk related to the toxic metals (*n* = 8). The RfD values used for Al, As, Ba, Cd, Co, Cu, Fe, Li, Mn, Ni, Pb, Se, and Zn were 1.0, 0.0003, 0.2, 0.0005, 0.0003, 1.50, 0.04, 0.70, 0.002, 0.14, 0.02, 0.0015, and 0.30 mg·kg^−1^ bw·day^−1^, respectively [64,65].

### 4.11. Daily Dietary Intakes

The daily dietary intakes (*DDIs %*) associated with the consumption of infusions of tea for adults (19 years and over) of chosen (according to the National Academy of Sciences, USA [66]) micro- and macroelements were calculated as follows:

*DDI* = *DI_i_*/*DRI_i_*, (4)

*DI_i_* = (*C_i_* × *IR*)/1000, (5)

where *DI_i_* is the daily intake, and *DRI_i_* is the dietary reference intake. The *DRI_i_* values calculated for Ca, Mg, Na, K, P, Cu, Fe, Mn, Se, and Zn were as follows: 1100, 317.5, 4700, 700, 1375, 8, 0.9, 13, 0.005, and 1.8 mg·day^−1^ for females and 1050, 415, 4700, 700, 1375, 0.035, 11, 0.9, 8, 0.005, and 2.3 mg·day^−1^ for males [66].

### 4.12. Statistical Analysis

ANOVA was applied to analyze the results. The significance of mean differences among the treatments was tested with the multiple comparison procedure, and Tukey’s range test was applied at a significance level of α = 0.05. The analysis was performed using the statistical software package Statistica v. 13.0 (StatSoft Inc., Tulsa, OK, USA). The ordination was calculated using the Canoco 5 program [67,68]. Principle component analysis (PCA) was used to determine the main trends in the data and to indicate the approximate direction of soil variable effects and the similarities and dissimilarities between location and treatments.

## 5. Conclusions

In this study, we tested wild and cultivated tea from different regions of China (Wuzhishan, Baisha, Kunlushan, and Pu’Er) to determine the concentrations of microelements, macrolements, nitrates, oxalates, and caffeine, as well as the antioxidant capacity and total phenolic content with regard to the health risks they may pose and to the nutritional requirements of adults. Our results showed that the tested teas differed significantly in terms of the concentration of selected macro- and microelements. In the case of micronutrients, higher concentrations were noted in dry material and infusions made from wild tea. The analysis indicated a greater affinity of wild tea for As, Al, Fe, Ni, Li, and Se; on the other hand, cultivated tea had a greater affinity for Cu and Zn. This was probably due to the much higher yield per unit area of tea grown on plantations, whereby the lower concentrations of micronutrients in cultivated tea may be related to the probable effect of microelement dilution in biomass. The analysis showed a greater affinity of wild tea for Mg and Na, while cultivated tea was more related to P and Ca. In the case of macronutrient content in the dry material, it was observed that significantly higher concentrations were associated with the use of NPK fertilizers in the studied plantations, while the differences in the content of macronutrients were also caused by the lower pH values characteristic for forest soils. The health risk indicators represented by target hazard quotient (THQ) and hazard index (HI) did not show that the tested teas present a direct health risk related to the concentration of evaluated elements. However, their concentration should be taken into account in terms of a comprehensive diet and in terms of long-term impact on human health. The analyzed teas can constitute a valuable source of Mn in the human diet. With respect to nitrates and oxalates, higher concentrations of these compounds were observed in cultivated tea fertilized with nitrogen. However, the concentrations of nitrates and oxalates did not exceed the safe level for daily intake. Nevertheless, because of higher concentrations of these compounds, cultivated tea could be considered as a more dangerous element of the diet. The analyzed cultivated tea can be characterized as a better source of antioxidants with a higher caffeine concentration.

## Figures and Tables

**Table 1:** Locations of sample collection.

Description	Type	Fertilizers	Pesticides	Mean Soil pH	Site	Coordinates
W1	Wild Tea	-	-	4.21	Wuzhishan	18°42′54.1″ N 109°36′03.5″ E
W2	Wild Tea	-	-	4.15	Baisha	19°03′39.3″ N 109°25′21.9″ E
W3	Wild Tea	-	-	4.45	Kunlushan	23°15′12.6″ N 101°03′49.2″ E
W4	Wild Tea	-	-	4.28	Pu’Er	22°43′06.6″ N 100°58′33.8″ E
C1	Cultivated Tea	N—310 kg·ha^−1^, P—70 kg·ha^−1^, K—105 kg·ha^−1^, zinc sulfate—1 kg Zn·ha^−1^	copper oxychloride—1.4 kg Cu·ha^−1^, deltamethrin—0.025 kg·ha^−1^, propiconazole—0.063 kg·ha^−1^.	4,79	Wuzhishan	18°42′34.2″ N 109°35′42.4″ E
C2	Cultivated Tea	N—300 kg·ha^−1^, P—80 kg·ha^−1^, K—110 kg·ha^−1^, zinc sulfate—1 kg Zn·ha^−1^	copper oxychloride—1.2 kg Cu·ha^−1^, thiacloprid—0.110 kg·ha^−1^, deltamethrin—0.025 kg·ha^−1^, propiconazole—0.063 kg·ha^−1^.	4.74	Baisha	19°11′13.0″ N 109°27′44.3″ E
C3	Cultivated Tea	N—300 kg·ha^−1^, P—86 kg·ha^−1^, K—115 kg·ha^−1^, zinc sulfate—1 kg Zn·ha^−1^	copper oxychloride—1.2 kg Cu·ha^−1^, deltamethrin—0.025 kg·ha^−1^, quinalphos—0.25 kg·ha^−1^, propiconazole—0.063 kg·ha^−1^.	4.82	Kunlushan	23°15′08.1″ N 101°04′10.6″ E
C4	Cultivated Tea	N—310 kg·ha^−1^, P—75 kg·ha^−1^, K—110 kg·ha^−1^, zinc sulfate—1 kg Zn·ha^−1^fertlizers	copper oxychloride—1.2 kg Cu·ha^−1^, deltamethrin—0.025 kg·ha^−1^, propiconazole—0.063 kg·ha^−1^.	4.65	Pu’Er	22°45′02.0″ N 100°59′30.1″ E

**Table 2:** Mean concentration of micro- and macroelements in dry tea material (mg·kg^−1^).

**Microelements**																				
	**Wild**								**Cultivated**								**Mean**			
	**Wuzhishan (W1)**		**Baisha (W2)**		**Kunlushan (W3)**		**Pu’Er (W4)**		**Wuzhishan (C1)**		**Baisha (C2)**		**Kunlushan (C3)**		**Pu’Er (C4)**		**Wild**		**Cultivated**	
	**mg·** **kg^−1^**	**SD**	**mg·** **kg^−1^**	**SD**	**mg·** **kg^−1^**	**SD**	**mg·** **kg^−1^**	**SD**	**mg·** **kg^−1^**	**SD**	**mg·** **kg^−1^**	**SD**	**mg·** **kg^−1^**	**SD**	**mg·** **kg^−1^**	**SD**	**mg·** **kg^−1^**	**SD**	**mg·** **kg^−1^**	**SD**
Al	6801.4 a*	847.59	6334.4 a	1578.94	5747.7 a	1977.28	6217.2 a	1617.92	5119.7 a	1739.27	3950.5 a	521.67	5367.6 a	1064.01	4509.3 a	2735.67	6275.24 B	473.80	4736.8 A	954.02
As	0.130 a	0.0479	0.177 a	0.1363	0.109 a	0.0402	0.194 a	0.1056	0.124 a	0.0318	0.137 a	0.0569	0.094 a	0.0265	0.082 a	0.0813	0.152 A	0.0462	0.109 A	0.0252
Ba	65.23 a	51.191	56.01 a	18.773	44.37 a	15.8338	46.73 a	20.194	104.51 a	14.491	53.85 a	7.0384	38.41 a	13.941	48.27 a	31.0440	53.090 A	16.562	61.28 A	10.190
Cd	0.168 a	0.0598	0.210 a	0.0649	0.154 a	0.0965	0.183 a	0.0648	0.328 a	0.0278	0.297 a	0.1638	0.119 a	0.0372	0.146 a	0.0624	0.179 A	0.0168	0.222 A	0.0624
Co	0.073 a	0.0948	0.160 ab	0.0920	0.102 ab	0.1087	0.118 ab	0.0901	0.147 ab	0.0645	0.286 b	0.1001	0.001 a	0.0401	0.033 a	0.0485	0.113 A	0.0084	0.117 A	0.0265
Cu	8.85 a	1.304	9.93 a	2.154	8.71 a	1.394	9.43 a	2.120	10.40 a	3.154	10.23 a	0.981	10.932 a	2.777	13.053 a	5.2273	9.235 A	0.456	11.15 B	1.742
Fe	72.12 a	18.914	116.22 a	35.552	140.94 a	56.035	111.78 a	58.821	70.12 a	12.739	72.61 a	12.606	87.433 a	17.133	70.293 a	113.138	110.270 B	18.744	75.11 A	49.534
Li	0.306 abc	0.0771	0.420 bc	0.1239	0.448 abc	0.1343	0.365 abc	0.1412	0.535 c	0.2498	0.276 abc	0.0935	0.113 a	0.0229	0.108 ac	0.2454	0.385 A	0.0289	0.258 A	0.1131
Mn	1730.1 a	361.58	1740.9 a	278.36	1407.9 a	608.97	1116.7 a	753.37	1909.7 a	726.28	1447.7 a	778.53	1299.25 a	347.19	1663.04 a	707.40	1498.95 A	219.36	1579.9 A	212.32
Ni	8.692 ab	1.1143	10.709 ab	4.3109b	6.898 ab	3.7010	9.308 b	5.1869	7.347 ab	0.6098	7.434 ab	1.8485	4.105 a	0.7595	4.220 a	1.7272	8.902 B	1.7522	5.777 A	0.6418
Pb	0.882 a	0.7724	0.494 a	0.1708	1.000 a	0.7416	0.622 a	0.2630	0.674 a	0.2054	0.716 a	0.2114	0.406 a	0.1161	0.549 a	0.4943	0.750 A	0.3144	0.587 A	0.1642
Se	0.454 c	0.015	0.377 ab	0.064	0.445 c	0.045	0.414 abc	0.080	0.368 a	0.029	0.404 b	0.010	0.411 abc	0.077	0.425 abc	0.068	0.422 B	0.022	0.402 A	0.025
Zn	8.994 a	0.8407	9.709 a	1.0501	10.069 a	1.1142	9.580 a	1.3579	8.406 a	0.3091	10.823 a	3.9846	29.551 a	4.5319	10.63 a	2.9795	9.58 A	0.213	10.14 B	0.475
**Macroelements**																				
	**Wild**								**Cultivated**								**Mean**			
	**Wuzhishan (W1)**		**Baisha (W2)**		**Kunlushan (W3)**		**Pu’Er (W4)**		**Wuzhishan (C1)**		**Baisha (C2)**		**Kunlushan (C3)**		**Pu’Er (C4)**		**Wild**		**Cultivated**	
	**mg·** **kg^−1^**	**SD**	**mg·** **kg^−1^**	**SD**	**mg·** **kg^−1^**	**SD**	**mg·** **kg^−1^**	**SD**	**mg·** **kg^−1^**	**SD**	**mg·** **kg^−1^**	**SD**	**mg·** **kg^−1^**	**SD**	**mg·** **kg^−1^**	**SD**	**mg·** **kg^−1^**	**SD**	**mg·** **kg^−1^**	**SD**
Ca	8869.3 ab	904.18	9754.6	722.72	8711.8 ab	874.92	9120.8 ab	1466.38	10491.7 ab	1240.86	7523.11 a	881.51	10985.1 b	1862.37	10415.7 ab	3797.23	9114.1 A	326.07	9939.3 A	1300.47
K	16780.9 a	3043.86	18319.2 a	7308.50	16207.9 a	2426.90	18398.8 a	6377.44	12127.5 a	3202.45	19163.3 a	2159.97	17856.9 a	3695.28	17113.8 a	8021.26	17426.7 A	2414.96	16676.8 A	2942.08
Mg	2810.5 a	438.08	3543.9 a	1562.37	2614.8 a	838.81	3147.9 a	1775.54	3433.6 a	889.18	2710.2 a	297.67	2725.1 a	278.63	2612.4 a	973.42	3029.0 A	623.16	2887.2 A	398.96
Na	87.99 a	27.092	122.97 a	254.786	70.66 a	25.914	89.48 a	37.011	99.74 a	20.572	94.56 a	19.478	68.91 a	26.008	62.96 a	41.598	92.78 B	19.770	82.43 A	10.601
P	898.9 a	300.34	935.3 a	261.00	1174.3 a	193.73	1185.5 a	121.36	1356.4 b	94.30	1488.5 b	124.39	1556.2 b	388.74	1687.9 b	1006.203	1048.5 A	229.17	1480.0 B	451.32

* Means with different letters within a row are significantly different, according to Tukey’s test (*p* ≤ 0.05); SD—standard deviation.

**Table 3:** Mean concentration of for macro- and microelements in tea infusions (mg·kg^−1^).

**Microelements**																				
**Wild**									**Cultivated**								**Mean**			
	**Wuzhishan (W1)**		**Baisha (W2)**		**Kunlushan (W3)**		**Pu’Er (W4)**		**Wuzhishan (C1)**		**Baisha (C2)**		**Kunlushan (C3)**		**Pu’Er (C4)**		**Wild**		**Cultivated**	
	**mg·** **kg^−1^**	**SD**	**mg·** **kg^−1^**	**SD**	**mg·** **kg^−1^**	**SD**	**mg·** **kg^−1^**	**SD**	**mg·** **kg^−1^**	**SD**	**mg·** **kg^−1^**	**SD**	**mg·** **kg^−1^**	**SD**	**mg·** **kg^−1^**	**SD**	**mg·** **kg^−1^**	**SD**	**mg·** **kg^−1^**	**SD**
Al	705.11 a*	197.40	686.98 a	200.13	742.89 a	169.97	756.31 a	181.02	626.50 a	300.90	539.57 a	55.38	542.58 a	190.61	663.05 a	59.53	722.82 B	14.22	592.92 A	117.69
As	0.039 a	0.0217	0.036 a	0.0055	0.034 a	0.0080	0.037 a	0.0100	0.035 a	0.0113	0.026 a	0.0092	0.034 a	0.0120	0.041 a	0.0119	0.037 A	0.0071	0.034 A	0.0013
Ba	3.555 ab	1.0721	3.805 a b	0.7711	3.663 a b	0.7150	3.256 a b	0.9490	5.086 b	1.4847	2.377 a	0.4413	1.669 a	0.6374	2.905 a	0.4271	3.570 B	0.1640	3.009 A	0.4006
Cd	0.017 a	0.0020	0.015 a	0.0050	0.018 a	0.0067	0.019 a	0.0057	0.023 a	0.0032	0.023 a	0.0036	0.016 a	0.0033	0.022 a	0.0021	0.017 A	0.0020	0.021 B	0.0007
Co	0.017 a	0.0173	0.036 a	0.0115	0.027 a	0.0223	0.031 a	0.0044	0.034 a	0.0114	0.056 b	0.0133	0.005 a	0.0004	0.007 a	0.0095	0.028 A	0.0077	0.025 A	0.0057
Cu	0.923 a	0.1389	1.255 a	0.5651	0.999 a	0.2309	1.223 a	0.4947	1.147 a	0.2193	1.145 a	0.2363	1.327 a	0.2106	1.645 a	0.0820	1.100 A	0.2047	1.316 A	0.0709
Fe	5.787 a	4.981	2.765 a	1.355	2.795 a	1.315	3.013 a	2.779	6.219 a	6.448	6.296 a	6.695	3.833 a	3.865	2.380 a	2.957	3.590 A	1.722	4.682 A	1.865
Li	0.087 a	0.0164	0.100 a	0.0276	0.080 a	0.0400	0.078 a	0.0301	0.141 b	0.0684	0.075 a	0.0273	0.027 a	0.0029	0.029 a	0.0445	0.086 A	0.0097	0.068 A	0.0277
Mn	207.76 a	75.67	183.64 a	57.70	213.278 a	147.64	281.64 a	86.65	311.20 a	100.77	274.02 a	35.54	83.19 a	39.47	184.64 a	24.93	221.58 A	39.02	213.26 A	34.28
Ni	2.485 ab	0.5495	3.027 c	1.3226	2.453 a c	0.7494	3.202 c	0.8351	2.220 a b	0.3377	2.174 a b	0.5098	1.052 a	0.2266	1.200 a	0.3541	2.792 B	0.3282	1.661 A	0.1165
Pb	0.102 a	0.0481	0.215 a	0.1603	0.202 a	0.1722	0.106 a	0.0376	0.137 a	0.0482	0.157 a	0.0910	0.104 a	0.0314	0.098 a	0.1473	0.157 A	0.0715	0.124 A	0.0517
Se	0.054 b	0.009	0.049 b	0.008	0.049 b	0.007	0.050 b	0.006	0.040 a	0.007	0.048 b	0.007	0.053 b	0.008	0.051 b	0.004	0.051 A	0.005	0.048 A	0.005
Zn	1.014 ab	0.3080	1.101 a b	0.3797	0.922 a	0.1443	1.070 a b	0.3342	0.969 b	0.1639	1.266 a b	0.4873	1.329 a b	0.4784	1.219 b	0.1551	1.027 A	0.1025	1.196 A	0.1868
**Macroelements**																				
	**Wild**								**Cultivated**								**Mean**			
	**Wuzhishan (W1)**		**Baisha (W2)**		**Kunlushan (W3)**		**Pu’Er (W4)**		**Wuzhishan (C1)**		**Baisha (C2)**		**Kunlushan (C3)**		**Pu’Er (C4)**		**Wild**		**Cultivated**	
	**mg·** **kg^−1^**	**SD**	**mg·** **kg^−1^**	**SD**	**mg·** **kg^−1^**	**SD**	**mg·** **kg^−1^**	**SD**	**mg·** **kg^−1^**	**SD**	**mg·** **kg^−1^**	**SD**	**mg·** **kg^−1^**	**SD**	**mg·** **kg^−1^**	**SD**	**mg·** **kg^−1^**	**SD**	**mg·** **kg^−1^**	**SD**
Ca	267.3 a	45.64	257.6 a	23.35	251.1 a	22.81	271.8 a	33.44	263.8 a	40.38	228.3 a	22.29	176.2 a	12.68	204.7 a	16.08	261.9 B	10.73	218.3 A	12.34
K	4314.8 a	871.45	4701.6 a	1910.94	4008.2 a	389.36	4772.3 a	1738.56	3395.7 a	953.08	4921.9 a	500.82	4603.5 a	637.85	4164.9 a	562.24	4449.2 A	720.54	4271.5 A	201.03
Mg	342.4 a	94.41	431.5 a	246.26	311.2 a	90.41	420.0 a	220.80	544.0 a	166.62	347.1 a	39.04	218.4 a	42.24	281.4 a	53.78	376.2 A	82.15	347.7 A	61.13
Na	35.52 a	17.874	50.93 a	24.750	29.19 a	19.029	37.29 a	22.352	43.81 a	2.535	39.83 a	7.220	27.28 a	3.049	27.32 a	2.127	38.24 A	7.776	34.56 A	2.356
P	61.02 a	7.490	76.63 a	45.598	73.25 a	26.575	78.76 a	37.081	92.70 b	29.227	186.92 b	24.500	198.80 b	32.052	250.98 b	20.595	72.42 A	16.424	182.35 B	5.070

* Means with different letters within row are significantly different, according to Tukey’s test (*p* ≤ 0.05); SD—standard deviation.

**Table 4:** Transfer coefficients for macro- and microelements.

**Microelements**																				
	**Wild**								**Cultivated**								**Mean**			
	**Wuzhishan (W1)**		**Baisha (W2)**		**Kunlushan (W3)**		**Pu’Er (W4)**		**Wuzhishan (C1)**		**Baisha (C2)**		**Kunlushan (C3)**		**Pu’Er (C4)**		**Wild**		**Cultivated**	
	**%**	**SD**	**%**	**SD**	**%**	**SD**	**%**	**SD**	**%**	**SD**	**%**	**SD**	**%**	**SD**	**%**	**SD**	**%**	**SD**	**%**	**SD**
Al	10.2 a*	1.72	10.9 a	1.98	11.5 a	1.62	10.9 a	1.62	11.9 a	2.02	13.7 a	1.01	10.2 a	2.67	11.0 a	0.89	10.9 A	2.41	11.7 A	1.99
As	35.4 a	23.92	30.2 a	19.57	34.9	15.39	24.6 a	16.83	29.4 a	11.67	26.4 a	24.75	37.2 a	25.64	43.9	48.33	31.3 A	15.46	39.3 A	13.06
Ba	7.7 a	4.81	7.7 a	3.71	7.5 a	4.12	7.0 a	4.42	4.8 a	0.92	4.5 a	1.08	5.1 a	3.68	6.1 a	0.59	7.5 A	0.94	5.1 A	0.77
Cd	11.0 ab	4.66	7.6 a	2.26	9.5 ab	5.49	8.0 a	1.22	6.9 a	0.58	9.4 ab	3.98	14.7 b	6.43	16.2 b	6.83	9.0 A	3.70	11.8 A	3.97
Co	24.7 a	20.91	18.9 a	4.89	19.8 a	11.98	21.9 a	8.05	24.1 a	4.15	20.2 a	3.83	23.2	5.22	21.9 a	12.76	21.3 A	3.22	22.3 A	4.32
																	A		A	
Cu	10.4 a	0.91	12.4 a	4.28	10.8 a	1.65	12.3 a	3.65	11.3 a	2.02	11.1 a	1.33	12.1 a	2.51	13.3 a	4.01	11.5 A	0.71	11.9 A	0.72
Fe	9.6 a	10.08	12.5 a	1.52	12.6 a	1.48	9.7 a	3.92	10.3 a	12.28	8.3 a	7.41	12.8 a	0.76	13.4 a	0.61	11.1 A	3.27	11.0 A	3.33
Li	28.9 a	2.56	23.9 a	2.82	24.7 a	3.47	28.6 a	2.74	26.2 a	1.86	27.0 a	3.95	23.9 a	6.41	29.0 a	11.04	26.5 A	2.21	26.6 A	1.83
Mn	8.4 a	3.61	9.5 a	2.81	8.3 a	1.49	12.3 a	5.87	13.2 a	6.32	10.0 a	3.42	6.7 a	2.38	7.9 a	3.52	9.6 A	0.98	9.8 A	1.22
Ni	28.3 a	3.11	27.7 a	4.30	27.2 a	3.48	29.7 a	2.05	30.1 a	2.19	29.4 a	2.64	27.3 a	2.75	28.2 a	1.98	28.3 A	0.97	28.7 A	1.23
Pb	17.7 a	11.87	19.9 a	34.56	21.1 a	21.32	19.8 a	10.74	21.9 a	11.62	21.5 a	8.77	25.8 a	10.06	18.3 a	5.66	19.6 A	13.27	21.9 A	13.33
Se	12.0 ab	1.11	13.0 b	1.62	11.0 a	1.63	12.0 ab	2.04	11.0 a	0.72	12.0 ab	1.21	13.0 ab	1.91	12.0 ab	1.72	10.3 A	2.61	12.0 A	1.64
Zn	11.3 a	3.20	11.2 a	3.19	9.7 a	1.23	11.6 a	2.49	11.5 a	1.51	11.7 a	0.77	11.1 a	3.50	12.0 a	2.35	10.9 A	0.35	11.6 A	0.29
**Macroelements**																				
**Wild**									**Cultivated**								**Mean**			
	**Wuzhishan (W1)**		**Baisha (W2)**		**Kunlushan (W3)**		**Pu’Er (W4)**		**Wuzhishan (C1)**		**Baisha (C2)**		**Kunlushan (C3)**		**Pu’Er (C4)**		**Wild**		**Cultivated**	
	**%**	**SD**	**%**	**SD**	**%**	**SD**	**%**	**SD**	**%**	**SD**	**%**	**SD**	**%**	**SD**	**%**	**SD**	**%**	**SD**	**%**	**SD**
Ca	3.0 a	0.62	2.7 a	0.39	2.6 a	0.54	2.8 a	0.78	2.5 a	0.11	3.1 a	0.44	1.6 a	0.19	2.0 a	0.38	2.8 B	0.36	2.3 A	0.16
K	25.6 a	1.07	25.8 a	2.17	25.7 a	2.12	26.6 a	0.55	27.9 a	1.04	25.7 a	1.39	22.9 a	2.07	25.0 a	2.81	25.9 A	0.80	25.4 A	0.78
Mg	12.2 a	1.79	12.2 a	2.02	11.9 a	2.01	13.3 a	0.41	15.8 a	1.89	12.8 a	0.91	7.6 a	1.82	10.8 a	0.93	12.4 A	0.77	11.7 A	0.54
Na	32.3 a	3.14	33.2 a	5.27	33.0 ab	12.78	33.3 ab	10.85	44.9 b	7.29	42.5 b	5.48	42.4 ab	16.70	40.3 ab	21.12	32.9 A	4.55	42.4 B	7.49
P	6.8 a	0.54	7.7 a	2.71	7.4 a	1.90	7.4 a	1.86	8.3 a	2.24	12.6 a	1.29	12.1 a	1.64	12.8 a	7.20	7.3 A	0.90	11.5 B	2.77

* Means with different letters within row are significantly different, according to Tukey’s test (*p* ≤ 0.05); SD—standard deviation.

**Table 5:** Estimated daily intakes (EDI) (mg·kg^−1^ bw·day^−1^) of micro- and macroelements for adults due to the consumption of tea.

**Microelements**										
**Wild**					**Cultivated**				**Mean**	
	**Wuzhishan (W1)**	**Baisha (W2)**	**Kunlushan (W3)**	**Pu’Er (W4)**	**Wuzhishan (C1)**	**Baisha (C2)**	**Kunlushan (C3)**	**Pu’Er (C4)**	**Wild**	**Cultivated**
Al	1.15 × 10^−1^	1.12 × 10^−1^	1.21 × 10^−1^	1.23 × 10^−1^	1.02 × 10^−1^	8.79 × 10^−2^	8.84 × 10^−2^	1.08 × 10^−1^	1.18 × 10^−1^	9.66 × 10^−2^
As	6.33 × 10^−6^	5.90 × 10^−6^	5.59 × 10^−6^	6.03 × 10^−6^	5.75 × 10^−6^	4.30 × 10^−6^	5.54 × 10^−6^	6.66 × 10^−6^	5.96 × 10^−6^	5.56 × 10^−6^
Ba	5.79 × 10^−4^	6.20 × 10^−4^	5.97 × 10^−4^	5.30 × 10^−4^	8.28 × 10^−4^	3.87 × 10^−4^	2.72 × 10^−4^	4.73 × 10^−4^	5.81 × 10^−4^	4.90 × 10^−4^
Cd	2.71 × 10^−6^	2.51 × 10^−6^	2.91 × 10^−6^	3.11 × 10^−6^	3.71 × 10^−6^	3.80 × 10^−6^	2.58 × 10^−6^	3.54 × 10^−6^	2.81 × 10^−6^	3.41 × 10^−6^
Co	2.75 × 10^−6^	5.88 × 10^−6^	4.41 × 10^−6^	5.03 × 10^−6^	5.50 × 10^−6^	9.10 × 10^−6^	7.72 × 10^−7^	1.20 × 10^−6^	4.52 × 10^−6^	4.14 × 10^−6^
Cu	1.50 × 10^−4^	2.04 × 10^−4^	1.63 × 10^−4^	1.99 × 10^−4^	1.87 × 10^−4^	1.86 × 10^−4^	2.16 × 10^−4^	2.68 × 10^−4^	1.79 × 10^−4^	2.14 × 10^−4^
Fe	9.42 × 10^−4^	4.50 × 10^−4^	4.55 × 10^−4^	4.91 × 10^−4^	1.01 × 10^−3^	1.03 × 10^−3^	6.24 × 10^−4^	3.88 × 10^−4^	5.85 × 10^−4^	7.62 × 10^−4^
Li	1.42 × 10^−5^	1.62 × 10^−5^	1.31 × 10^−5^	1.26 × 10^−5^	2.29 × 10^−5^	1.22 × 10^−5^	4.38 × 10^−6^	4.74 × 10^−6^	1.40 × 10^−5^	1.11 × 10^−5^
Mn	3.38 × 10^−2^	2.99 × 10^−2^	3.47 × 10^−2^	4.59 × 10^−2^	5.07 × 10^−2^	4.46 × 10^−2^	1.35 × 10^−2^	3.01 × 10^−2^	3.61 × 10^−2^	3.47 × 10^−2^
Ni	4.05 × 10^−4^	4.93 × 10^−4^	3.99 × 10^−4^	5.21 × 10^−4^	3.61 × 10^−4^	3.54 × 10^−4^	1.71 × 10^−4^	1.95 × 10^−4^	4.55 × 10^−4^	2.71 × 10^−4^
Pb	1.67 × 10^−5^	3.50 × 10^−5^	3.30 × 10^−5^	1.73 × 10^−5^	2.23 × 10^−5^	2.55 × 10^−5^	1.69 × 10^−5^	1.59 × 10^−5^	2.55 × 10^−5^	2.02 × 10^−5^
Se	8.86 × 10^−6^	7.97 × 10^−6^	7.97 × 10^−6^	8.09 × 10^−6^	6.58 × 10^−6^	7.89 × 10^−6^	8.69 × 10^−6^	8.31 × 10^−6^	8.22 × 10^−6^	7.87 × 10^−6^
Zn	1.65 × 10^−4^	1.79 × 10^−4^	1.50 × 10^−4^	1.74 × 10^−4^	1.58 × 10^−4^	2.06 × 10^−4^	2.16 × 10^−4^	1.99 × 10^−4^	1.67 × 10^−4^	1.95 × 10^−4^
**Macroelements**										
	**Wild**				**Cultivated**				**Mean**	
	**Wuzhishan (W1)**	**Baisha (W2)**	**Kunlushan (W3)**	**Pu’Er (W4)**	**Wuzhishan (C1)**	**Baisha (C2)**	**Kunlushan (C3)**	**Pu’Er (C4)**	**Wild**	**Cultivated**
Ca	4.35 × 10^−2^	4.20 × 10^−2^	3.51 × 10^−2^	4.09 × 10^−2^	4.30 × 10^−2^	3.72 × 10^−2^	2.87 × 10^−2^	3.34 × 10^−2^	3.56 × 10^−2^	4.43 × 10^−2^
Mg	7.03 × 10^−1^	7.66 × 10^−1^	7.31 × 10^−1^	6.53 × 10^−1^	5.53 × 10^−1^	8.02 × 10^−1^	7.50 × 10^−1^	6.78 × 10^−1^	6.96 × 10^−1^	7.77 × 10^−1^
Na	7.25 × 10^−2^	9.14 × 10^−2^	5.23 × 10^−2^	6.59 × 10^−2^	8.86 × 10^−2^	5.65 × 10^−2^	3.56 × 10^−2^	4.58 × 10^−2^	5.66 × 10^−2^	8.89 × 10^−2^
K	1.16 × 10^−2^	1.66 × 10^−2^	4.86 × 10^−3^	9.51 × 10^−3^	7.14 × 10^−3^	6.49 × 10^−2^	4.44 × 10^−3^	4.45 × 10^−3^	5.63 × 10^−3^	1.21 × 10^−2^
P	9.94 × 10^−3^	1.25 × 10^−2^	1.56 × 10^−2^	1.19 × 10^−2^	1.51 × 10^−2^	3.04 × 10^−2^	3.24 × 10^−2^	4.09 × 10^−2^	2.97 × 10^−2^	1.28 × 10^−2^

**Table 6:** The calculated target hazard quotients (THQs) of microelements and the accumulative hazard indices (HI) for adults associated with the consumption of infusions of tea.

	Wild				Cultivated				Mean	
	Wuzhishan (W1)	Baisha (W2)	Kunlushan (W3)	Pu’Er (W4)	Wuzhishan (C1)	Baisha (C2)	Kunlushan (C3)	Pu’Er (C4)	Wild	Cultivated
Al	1.15 × 10^−1^	1.12 × 10^−1^	1.21 × 10^−1^	1.23 × 10^−1^	1.02 × 10^−1^	8.79 × 10^−2^	8.84 × 10^−2^	1.08 × 10^−1^	9.66 × 10^−2^	1.18 × 10^−1^
As	2.11 × 10^−2^	1.97 × 10^−2^	1.86 × 10^−2^	2.01 × 10^−2^	1.92 × 10^−2^	1.43 × 10^−2^	1.85 × 10^−2^	2.22 × 10^−2^	1.85 × 10^−2^	1.99 × 10^−2^
Ba	2.89 × 10^−3^	3.10 × 10^−3^	2.98 × 10^−3^	2.65 × 10^−3^	4.14 × 10^−3^	1.94 × 10^−3^	1.36 × 10^−3^	2.37 × 10^−3^	2.45 × 10^−3^	2.91 × 10^−3^
Cd	5.42 × 10^−3^	5.02 × 10^−3^	5.82 × 10^−3^	6.22 × 10^−3^	7.43 × 10^−3^	7.61 × 10^−3^	5.16 × 10^−3^	7.07 × 10^−3^	6.82 × 10^−3^	5.62 × 10^−3^
Co	9.16 × 10^−3^	1.96 × 10^−2^	1.47 × 10^−2^	1.68 × 10^−2^	1.83 × 10^−2^	3.03 × 10^−2^	2.57 × 10^−3^	4.02 × 10^−3^	1.38 × 10^−2^	1.51 × 10^−2^
Cr	3.90 × 10^−5^	4.64 × 10^−5^	3.76 × 10^−5^	5.91 × 10^−5^	2.67 × 10^−5^	1.22 × 10^−5^	5.10 × 10^−5^	2.31 × 10^−5^	2.83 × 10^−5^	4.56 × 10^−5^
Cu	3.76 × 10^−3^	5.11 × 10^−3^	4.07 × 10^−3^	4.98 × 10^−3^	4.67E × 10^−3^	4.66 × 10^−3^	5.40 × 10^−3^	6.70 × 10^−3^	5.36 × 10^−3^	4.48 × 10^−3^
Fe	1.35 × 10^−3^	6.43 × 10^−4^	6.50 × 10^−4^	7.01 × 10^−4^	1.45E × 10^−3^	1.46 × 10^−3^	8.92 × 10^−4^	5.54 × 10^−4^	1.09 × 10^−3^	8.35 × 10^−4^
Li	7.10 × 10^−3^	8.12 × 10^−3^	6.53 × 10^−3^	6.32 × 10^−3^	1.15 × 10^−2^	6.10 × 10^−3^	2.19 × 10^−3^	2.37 × 10^−3^	5.53 × 10^−3^	7.02 × 10^−3^
Mn	2.42 × 10^−1^	2.14 × 10^−1^	2.48 × 10^−1^	3.28 × 10^−1^	3.62 × 10^−1^	3.19 × 10^−1^	9.68 × 10^−2^	2.15 × 10^−1^	2.48 × 10^−1^	2.58 × 10^−1^
Ni	2.02 × 10^−2^	2.47 × 10^−2^	2.00 × 10^−2^	2.61 × 10^−2^	1.81 × 10^−2^	1.77 × 10^−2^	8.57 × 10^−3^	9.77 × 10^−3^	1.35 × 10^−2^	2.27 × 10^−2^
Pb	1.11 × 10^−2^	2.33 × 10^−2^	2.20 × 10^−2^	1.16 × 10^−2^	1.49 × 10^−2^	1.70 × 10^−2^	1.13 × 10^−2^	1.06 × 10^−2^	1.34 × 10^−2^	1.70 × 10^−2^
Se	1.77 × 10^−3^	1.59 × 10^−3^	1.59 × 10^−3^	1.62 × 10^−3^	1.32 × 10^−3^	1.58 × 10^−3^	1.74 × 10^−3^	1.66 × 10^−3^	1.65 × 10^−3^	1.57 × 10^−3^
Zn	5.51 × 10^−4^	5.98 × 10^−4^	5.01 × 10^−4^	5.81 × 10^−4^	5.26 × 10^−4^	6.87 × 10^−4^	7.21 × 10^−4^	6.62 × 10^−4^	6.49 × 10^−4^	5.57 × 10^−4^
HI (hazard index) = THQ1 + THQ2 +	0.44 b	0.44 b	0.47 b	0.55 c	0.56 c	0.51 bc	0.25 a	0.39 b	0.43 A	0.47 A

* Means with different letters within row are significantly different, according to Tukey test (p ≤ 0.05).

**Table 7:** Daily dietary intakes (%) associated with the consumption of infusions of tea for adults (19 years and over) of micro- and macroelements with regard to dietary reference intakes (DRIs).

**Microelements**																				
	**Wild**								**Cultivated**								**Mean**			
	**Wuzhishan (W1)**		**Baisha (W2)**		**Kunlushan (W3)**		**Pu’Er (W4)**		**Wuzhishan (C1)**		**Baisha (C2)**		**Kunlushan (C3)**		**Pu’Er (C4)**		**Wild**		**Cultivated**	
	M *	F **	**M**	**F**	**M**	**F**	**M**	**F**	**M**	**F**	**M**	**F**	**M**	**F**	**M**	**F**	**M**	**F**	**M**	**F**
																				
Zn	0.11	0.14	0.11	0.16	0.12	0.13	0.11	0.15	0.1	0.14	0.13	0.18	0.14	0.19	0.13	0.17	0.11	0.15	0.12	0.17
Cu	1.17	1.17	1.59	1.59	1.26	1.26	1.55	1.55	1.45	1.45	1.45	1.45	1.68	1.68	2.08	2.08	1.39	1.39	1.67	1.67
Fe	0.82	0.6	0.39	0.28	0.4	0.29	0.43	0.31	0.89	0.68	0.9	0.65	0.55	0.39	0.34	0.24	0.51	0.37	0.67	0.49
Mn	102.98	131.58	91.02	116.31	105.71	135.08	139.59	178.37	154.25	197.09	135.82	173.55	41.23	52.69	91.52	116.94	109.83	140.33	105.7	135.07
Se	0.016	0.016	0.014	0.014	0.014	0.014	0.015	0.015	0.012	0.012	0.014	0.014	0.016	0.016	0.015	0.015	0.015	0.015	0.014	0.014
**Macroelements**																				
	**Wild**								**Cultivated**								**Mean**			
	**Wuzhishan (W1)**		**Baisha (W2)**		**Kunlushan (W3)**		**Pu’Er (W4)**		**Wuzhishan (C1)**		**Baisha (C2)**		**Kunlushan (C3)**		**Pu’Er (C4)**		**Wild**		**Cultivated**	
	**M**	**F**	**M**	**F**	**M**	**F**	**M**	**F**	**M**	**F**	**M**	**F**	**M**	**F**	**M**	**F**	**M**	**F**	**M**	**F**
Ca	0.29	0.28	0.28	0.27	0.27	0.26	0.3	0.28	0.29	0.27	0.25	0.24	0.19	0.18	0.22	0.21	0.29	0.27	0.24	0.23
Mg	1.22	1.6	1.54	2.01	1.11	1.45	1.5	1.96	1.5	1.94	0.95	1.25	0.6	0.78	0.77	1.01	1.34	1.76	0.96	1.25
Na	0.06	0.06	0.09	0.09	0.02	0.02	0.05	0.05	0.04	0.04	0.03	0.03	0.02	0.02	0.02	0.02	0.03	0.03	0.06	0.06
K	1.05	1.05	1.14	1.14	0.97	0.97	1.16	1.16	0.82	0.82	1.19	1.19	1.12	1.12	1.01	1.01	1.08	1.08	1.04	1.04
P	0.1	0.1	0.12	0.12	0.12	0.12	0.13	0.13	0.15	0.15	0.3	0.3	0.32	0.32	0.41	0.41	0.12	0.12	0.3	0.3

* Male, ** Female.

**Table 8:** Parameters of the analysis method.

Parameters	Wavelengths	Limit of Detection	Content in Certificated Material	Measured	Recovery
	(nm)	(mg·dm^−3^)	(mg·kg^−1^)	(mg·kg^−1^)	(%)
Al **	396.153	0.0315	685	715.9	104.5
As *	188.979	0.0134	-	-	-
Ba	233.527	0.0042	6	5.694	94.9
Cd	228.802	0.0027	0.03	0.0311	103.7
Co	228.616	0.007	0.13	0.128	98.5
					
Cu	327.393	0.0097	9.4	9.752	103.7
Fe	238.204	0.0046	185	172.3	93.1
Li *	670.784	0.0826	-	-	-
Mn	257.608	0.0014	47	45.84	97.5
Ni	231.604	0.0151	4	3.89	97.3
Pb	220.353	0.0425	1.6	1.544	96.5
Se	196.323	0.0009	0.022	0.02485	112.95
Zn	206.200	0.0059	24	23.11	96.3
Ca	317.933	0.01	21,600	22,328	103.4
Mg	285.208	0.0016	1360	1320	97.1
Na	589.592	0.069	500	482	96.4
K	766.490	-	21,000	20,353	96.9
P	213.617	0.076	2300	2199	95.6

* The element is not included in the reference material. ** Internal tea reference material.

## Data Availability

Not applicable.
